# Best practices for effective implementation of online teaching and learning in medical and health professions education: during COVID-19 and beyond

**DOI:** 10.3934/publichealth.2022019

**Published:** 2022-01-27

**Authors:** Pradeep Kumar Sahu, Hakki Dalcik, Cannur Dalcik, Madan Mohan Gupta, Vijay Kumar Chattu, Srikanth Umakanthan

**Affiliations:** 1 Centre of Medical Sciences Education, Faculty of Medical Sciences, The University of the West Indies, St. Augustine, Trinidad and Tobago; 2 Faculty of Medicine, Istanbul Aydin University, Florya Istanbul, Turkey; 3 School of Pharmacy, Faculty of Medical Sciences, The University of the West Indies, St. Augustine, Trinidad and Tobago; 4 Department of Medicine, Temerty Faculty of Medicine, University of Toronto, Toronto, ON M5G 2C4, Canada; 5 Center for Transdisciplinary Research, Saveetha Institute of Medical and Technological Sciences, Saveetha University, Chennai, India; 6 Department of Community Medicine, Faculty of Medicine, Datta Meghe Institute of Medical Sciences, Wardha, India; 7 Department of Paraclinical Sciences, Faculty of Medical Sciences, The University of the West Indies, St. Augustine, Trinidad and Tobago

**Keywords:** medical education, health professions, online teaching, COVID-19, synchronous, asynchronous, assessment, technology

## Abstract

The COVID-19 pandemic has caused worldwide disruption to the entire educational system, including medical and health professions education. Considering the critical situation due to COVID-19, academic institutions shifted the entire pedagogical approach to the virtual learning mode. While delivering online teaching, educators experienced numerous challenges, including access to the internet, poor connectivity, and other technical issues. Some students did not have laptops and necessary devices to attend the Class. Besides, many educators were not confident enough to manage the online mode of delivery. In this perspective, we reviewed the evidence of best practices for the medical and health professions educators to deliver the curriculum through an online platform. Therefore, the current study aimed to review the best practices for effective online teaching and learning in medical and health professions education during COVID-19 and beyond. We reviewed the technical aspects of online teaching and educational strategies required for educators to provide quality training not just during the pandemic but beyond this crisis. The online literature search was performed on Medline, PubMed and google scholar databases for studies on online teaching in medical and health profession education and what are the best practices of teaching globally Online teaching and assessment must balance the requirements of technology, learning outcomes, delivery modes, learning resources, and learning resources. The study concludes that medical and health professions institutions strengthen technical infrastructure, promote continuous faculty development programs, and support indigent students to access digital technology.

## 1. Introduction

The COVID-19 pandemic has created a global health care crisis with a range of unprecedented challenges, including a severe impact on the entire education system. Medical and health education has been dramatically affected by the closure of higher education institutions. The effects of the COVID-19 pandemic are ongoing, and there is uncertainty about how long it will last. Considering the need of the situation, Universities across the globe prefer continuing teaching and learning via online mode [1]. Although online learning is not a new concept, it was not mandatory for academic institutions before the pandemic [2]. Medical teachers and students started learning how to equip themselves with technological skills. For some teachers, the immediate shifting from face-to-face to online teaching was a huge challenge, while others with prior experience quickly acclimatized themselves with this new learning mode [3]. Stoehr et al. (2021) found a positive attitude of medical students towards online learning. However, the study revealed a considerable discrepancy between what students demand and the curriculum offers. Thus, it was concluded by the authors that the COVID-19 pandemic might be the long-awaited catalyst for a new “online era” in medical education [4].

Globally, different medical schools were already using the learning management system (LMS) as a platform where the teachers were sharing the content, learning resources, assignments for their students [5],[6]. For both students and teachers, LMS was one of the effective online modes of communication. LMS, during the pandemic, eases the transition to online learning for educators of various medical schools [7].

The success or failure of online learning programs depends on the quality of technological infrastructure, necessary technical skills among teachers and students, access to the internet, and availability of computers, laptops, and required devices by the teachers and students [8],[9]. All medical institutions do not have a well-connected technological support system, and all students do not have equal access. Some students and teachers may not have a home environment conducive to teaching and learning. Along with the technology and IT support, medical teachers need to play a significant role in preparing academic strategy and educational design to deliver online teaching effectively. Some other challenges to online education are students' assessment, lack of interaction, lack of motivation, and time management [10]. To develop an effective online learning program and improve the quality of learning, there is a need to consider these barriers to online learning [11].

In this perspective, we reviewed the evidence of best practices for the medical educators to deliver the curriculum through an online platform. Therefore, the current study aimed to review the best practices for effective online teaching and learning in Medical Education during COVID-19 and beyond. We reviewed the technical aspects of online teaching and educational strategies required for educators to provide quality training during the pandemic and beyond this crisis.

Research questions of the study:

1. What knowledge and experiences are required for the faculty and students in health professions to adopt online teaching?
2. What infrastructure is required for online teaching?
3. What adjustments are required to curriculum planning and development when to deliver online teaching in medical and health professions education?
4. What methods are employed to evaluate the performance of medical and health professional students in online modes of teaching?

## 2. Methodology

In this review article, the search was focused on the literature on online teaching and learning in the context of medical and allied health sciences education. Medline, PubMed, and Google scholar were search engines where articles relevant to the medical and health profession were selected. This review includes various practices on how online teaching could be more impactful and effective during this COVID-19 situation and even in the future through distance education.

A systematic search was performed using the keywords: COVID-19, online learning, e-learning, blended learning, online teaching, innovative teaching, medical teaching, health profession teaching, web-based teaching, distance teaching and learning, learning outcomes, and online technology, assessment, Asynchronous assessment. The database searches generated a total of 105 articles. The authors read the full articles, discussed how to categorize them, and eventually, 53 articles were selected as relevant to best practices in medical and health profession education. These selected articles were grouped into four major categories—online teaching, technological infrastructure, innovative teaching practice, learning outcomes, and evaluation methods concerning the best practice of teaching and learning.

## 3. Theoretical framework for online learning

An understanding of learning theories can assist medical teachers in designing and implementing an effective online learning environment, and it will help them make choices about how to approach their teaching in ways that will best fit the needs of the students. There are three broad theoretical approaches such as behaviourism, cognitivism, and constructivism that have contributed to human learning and might have implications for online learning design [11].

According to behavioural learning theory influenced by Thorndike, Pavlov, and Skinner, learning is an observable change in a learner's behaviour caused by external stimuli. Behaviourists claim that practice or repetition of behaviour strengthens the link between stimuli and response [12]. Ignoring the impact of the cognitive or thinking process of the learner, this theory believes that learning occurs by practice and reinforcement. According to behaviourists, the role of the environment, specifically how stimuli are arranged and presented and how responses are reinforced, is of most importance. Early computer learning systems and programs instructions were designed based on a behaviourist approach to learning. Medical educators should provide feedback to the learner during online teaching as it assists the development of positive behaviour. Cognitive learning theory criticized the idea that learners are passive and simply react to stimuli in the environment [13].

Cognitive learning theory claims that learning is an internal mental phenomenon rather than the external environment involving memory, motivation, perceptions, critical thinking, and reflection [14],[15]. The cognitivist approach develops learners' capacity and skills for effective self-directed learning. According to this approach, the teacher facilitates the learner about “learn how to learn” [16]. Small group, online platforms should be provided to the learners to utilize their cognitive abilities. Also, online Problem Based Learning (PBL) is directly associated with cognitive learning theory where tutor facilitates a small group of learners for self-directed learning. Learners utilize their memories and cognitive abilities and think critically to construct new knowledge. The tutor, as a facilitator, ensures substantial contributions of all the learners in the group in the PBL approach.

Constructivist learning theories argue that learning is an active, contextualized process of constructing knowledge where learners are the learning centre. The constructivists believe that deep learning will occur through the active engagement of learners [17]. Even though constructivism is considered a branch of cognitivism (both focus on mental activity), it distinguishes itself from cognitivism in various ways. Cognitivism places the mind at the centre of the learning process, whereas constructivists believe that the mind filters input from the world to construct knowledge. According to constructivist learning theories, a learner's knowledge is assembled through exploration where his/her direct experiences with the environment are considered critical [18]. In constructivist psychology, learners are given control of learning and reflection. In online teaching, teamwork, collaborative and cooperative learning should be encouraged to facilitate constructivist learning. Assignments and projects should be given to the learners to help them apply and personalize the information. Small group teaching, PBL, and flipped classroom are effective online delivery modes for the learners.

Each school of learning offers benefits and limitations to the design and development of online learning. Medical educators should consider these theories while designing an online learning environment curriculum. Online learning pedagogy, which has been widely introduced during the pandemic, is now considered a crucial component in medical education [19]. A properly planned, structured, and dedicated online teaching curriculum would benefit the students. The current situation might present a unique opportunity for augmenting the quality of medical education to fulfil students' teaching needs [4].

## 4. Best practices for online teaching

In this section, the results are presented concerning the research questions.

### 4.1. Faculty and students must be familiar with online technology

The transition to online teaching has raised questions for the faculty about their capability to deal with the existing technology. Without adequate technical knowledge and skills, medical teachers/instructors may face numerous challenges to resolve technology-related problems during the live Class, impacting students' online learning and access to learning materials [20]. You should know about switching the microphone and camera into mute and unmute mode. Also, you can record and upload lectures for later viewing by students who were unable to attend or had internet or other problems [21]. Therefore, proper training and support services should be facilitated to the faculty to deal with technology efficiently. Students also must go through similar training to utilize the best of online teaching [22]. Initially, while using technology, you may experience some difficulties, but you will be confident using the platform after getting used to it. For example, finding and uploading the course materials, using the chat rooms, initiating, recording the sessions, and other activities will be more accessible for you.

### 4.2. Technological infrastructure and access to the internet

The successful functioning of online teaching depends on the availability of technological infrastructure in hardware and software [23]. The existing infrastructure in the medical schools is not adequate to run the various programs in online mode. Especially in the low-middle-income countries, lack of infrastructure and technology have become an obstacle to effectively implementing online teaching. These technological gaps are variable in different geographical regions where some countries lack the basics such as email internet (wired and wireless). At the same time, some are related to poor quality of the services such as poor/intermittent internet access and limited access to computers or laptops [9]. Network connectivity and bandwidth availability are the key challenges even in developed countries. The technological infrastructure and user-friendliness of the LMS played important roles in the online learning of medical and health professional students.

Few suggestions to address these problems are:

- Medical institutions should establish an ICT committee to develop strategies for accelerating a digital infrastructure to support and drive online teaching and assessment.
- Needy students should borrow laptops from the University Library to attend online teaching.
- Institutions should focus on digital infrastructure liaising with IT firms.
- IT firms should provide a compatible package for students to avail high internet speed for online classes.
- Students having difficulties accessing the internet should be allowed to attend the online Class.

Medical teachers/tutors who prefer to deliver teaching on the campus should be provided good internet connection. Technicians should also be available during live classes to avoid disruption or delay due to any technical issues [24].

### 4.3. Make sure that learning outcomes, teaching activities, and assessments are aligned

**Figure 1.**
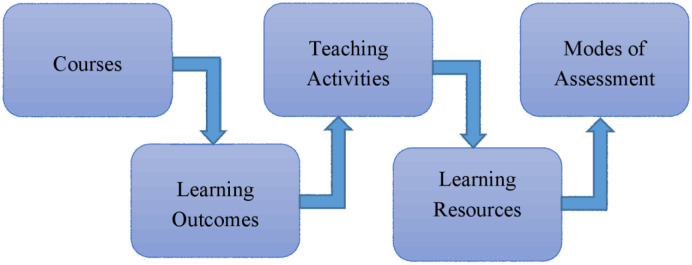
Course alignment for online teaching.

In online teaching and learning, clear expectations of what is required from students are critical. As a medical teacher, you should make sure that students know the course's learning outcomes before the Class begins. Furthermore, clearly defined learning outcomes help you to select different teaching activities and identify valid assessment tools to evaluate student's performance [25],[26]. Making learning trajectories explicit and visible for both students and teachers can help promote reflection and potentially enhance the teaching and learning process [27]. Proper alignment of learning outcomes, teaching activities, learning resources, and assessment modes mutually reinforce each other. While framing the learning outcomes, you can use Bloom's lists of action verbs or different authors' extended and revised action verbs [28]. Based on the learning outcomes, you should decide the learning activities and determine the teaching aids to be used in the online Class. Assessment criteria should be designed that meet the specific and measurable learning outcomes. Figure 1 shows the essential components of course alignment for online teaching.

The assessment method in medical and health profession education could be administered in an asynchronous mode during the COVID-19 pandemic. Assessment methods such as open-ended short answer questions, problem-based questions, oral exams, and recorded objective structured clinical exams (OSCE) would be appropriate for Use in an asynchronous environment to assess the knowledge and competence of health professional students during COVID-19.

### 4.4. Plan and prepare for the lesson before the class begins

The transition of the curriculum delivery from face-to-face teaching to online learning requires a lot of planning and preparation [29]. Even experienced medical teachers do not enter the Class without proper planning. You must have the answers to the questions, what your students would be able to know or do from the Class (instructional objectives), which area of the curriculum will be covered (content), what are the learning resources (teaching aids), how the content will be covered in the given time (time management), how the content can effectively be delivered (teaching method) and how the effectiveness of the teaching will be known (feedback and evaluation). Prepare a lesson plan for online teaching. Table 1 shows an example of a lesson plan on a few topics in medicine.

Similarly, you have to prepare lesson plans for the courses you are allotted for teaching. It will help you to understand how much you can fit into the session in the allocated time [30]. Once you have the lesson plan, you prepare for the teaching accordingly and make sure that you have all the PPT, images, videos, quizzes, questions available to share during the online teaching.

**Table 1.** Example of a lesson plan.

Course code and name	Name of the instructor/lecturer/tutor: XXX			
Topic and date	Instructional objectives	Teaching aids	Teaching method	Assessment
Cell injury and death Date: Duration:	-Discuss the types of cell death. -List the factors that are associated with cell growth and regeneration	-PPT -Recorded lectures -Interactive online materials	-Lecture -Case presentation -PBL	-Asynchronous clerkship assessment: MCQ format. -Synchronous clerkship assessment: museum specimen case presentations -In-class Online synchronous quiz and polls. -PBL Assessment: case-based study
Inflammation Date: Duration:	-Outline the differences between acute and chronic inflammation. -Explain the stages of acute inflammation with an emphasis on the inflammatory cells in each phase.	-Recorded lectures -Video clippings -Group based resource study -Guest lecture links.	-Lecture -Video demonstration -PBL	-Asynchronous clerkship assessment: Histology spotters -Synchronous clerkship assessment: short case discussion -Asynchronous Short answer essay questions -PBL assessment through objective-based presentation
Neoplasm Date: Duration:	-Discuss the morphological differences between benign and malignant tumors. -Outline in detail the features of metastasis.	-PPT -Recorded lectures -Question banks -Interactive online materials	-Lecture -Case presentation -PBL	-Asynchronous clerkship assessment: MCQ format. -Synchronous clerkship case presentations -PBL Assessment -Clinical Case reviews

### 4.5. Ensure flexible approach in teaching and have a backup plan

The transition to an online mode of delivery is not free from challenges, and poor technological infrastructure and network connectivity issues interrupt smooth course content delivery. Thus, you should have a flexible approach in teaching, assuming that at any time, teachers or students may have a problem with internet connectivity during the teaching session [31]. Sometimes, assignments and resources related to the course that teachers post do not work properly [32]–[34].

It would help if you always had plan B ready for all course contents, assignments, and assessments to overcome the technological glitches. For example, if you are delivering a lecture via Blackboard Collaborate and it stops working, the group should use Zoom or any other links without causing any learning delay. Similarly, students should be given extra time to submit their assignments and projects [31]. To make online teaching more effective, medical schools should provide a clear policy guideline that directs what students can do if they cannot submit assignments due to technical issues like electricity failure, poor internet connectivity, software disrupts, and computer system failure.

### 4.6. Exhibit strong virtual presence

In a face-to-face class, you can read students' moods their non-verbal communication and engage them in different activities to make an active and interactive classroom session. It is easy for you to motivate the less participatory student by involving them in group discussions. However, it is quite difficult for a teacher to motivate and engage all students in the Class [34]–[39]. To make the online synchronous Class more effective, you can do the following things:

- Before the Class starts, send reading material and video links to students.
- Welcome students to online Class by sending a video message.
- Do the learning activities through live video and/or audio conferencing with immediate feedback.
- During the Class, ask questions to students by calling them in their names so that students will remain active.
- While using technology like Blackboard Collaborate (BBC), use the whiteboard option to explain complex things by drawing pictures, diagrams, graphs, etc.
- To involve students in discussion, breakout them into small groups and give them topics for discussion.
- Additionally, teachers can use non-verbal communication such as chatting and emoji.
- Conduct a separate online revision or doubt-clear Class for the students.

### 4.7. Balance between synchronous and asynchronous learning

The hybrid online learning model includes synchronous and asynchronous learning. Synchronous learning happens in real-time. However, asynchronous learning happens online without real-time interaction [40]. There are advantages and disadvantages of both learning models. It is crucial to balance and use each one of them equally. Because among the students there may be different types of learners, such as socially active/passive, understand better/bad or do better/bad in a real-time virtual place? Some students might not desire to be at a specific place at a particular time in the videoconferencing, while others may. Some may be active learners getting immediate feedback from the teachers, and some may not. Some others might learn better and thrive if their questions are answered promptly. For some, it may not be necessary. Some students may want to interact with the teacher and receive mentorship, guidance, or support, while others may not. If there is too much asynchronous learning, the student may have difficulty coping with set schedules and fully depreciating their responsibilities. Therefore, among the students, some may prefer synchronous mode, whereas others may be comfortable with asynchronous learning mode. Considering the preference of both synchronous and asynchronous delivery modes would be the best option.

Face-to-face Lab or practical education is crucial in medical education. However, in terms of Anatomy labs, it is possible to demonstrate the cadavers, bones, or models synchronously with the appropriate camera settings and using online platforms. There is feedback obtained by many students that show that practical classes like Anatomy can be taught quite well online and can improve student performance [41]. An advanced 3D anatomy platform can also be used online to assist students in understanding the concept.

Although hands-on cadaver dissection holds a crucial part of Anatomy education, web-based synchronous cadaver dissection online recordings are the method that can be used. Similar studies have demonstrated web-based surgical skill learning sessions, and their skills were comparable to those taught by conventional face-to-face tutorials [42]. Further, online teaching attempts to substitute hands-on education due to pandemics, including demonstrations of practical procedures, remote patient consultation programs, and simulated cases [43].

### 4.8. Make the class interactive

The success of online learning largely depends on the active participation of all the students in the Class. An online class should be more interactive, and it should create a suitable environment for learning that is active, innovative, and challenging for the students [44]. Rice (2006) found that online teaching strategies make the best Use of the unique potential of the online environment when they are highly interactive [45]. An interactive approach makes the learning process effective and exciting and allows the teachers to assess students' performance spontaneously and non-intrusively. Students may be less active in the large group, and however, it becomes easier for teachers to engage students in the learning process in a small group. Instead of dominating the group, an educator promotes student-centred learning and actively encourages them to participate in the learning session. While using power-point, make sure that students get a chance to express their views and interact with something on every single slide.

To engage students, teachers should ask thought-provoking questions instead of yes/no type questions to think and try to take part in the discussion. Students should be encouraged to ask questions to drive them to be more attentive and active. Similarly, other students should be encouraged to respond to the questions asked by their peers. Teachers must appreciate students who are asking questions or responding to the questions. To improve the students' engagement with online sessions, online teaching should include quizzes and tests for the students, and it will help to analyse how actively students were involved in the learning process.

### 4.9. Timely feedback and motivation

One of the crucial risks of online education is cheating or unwanted students' behaviour while using online technology [46]. Compared with the traditional classroom settings, teachers have less control over the online Class, and students show the slightest interest in taking in the teaching-learning process [47]. Students' motivation is one of the significant determinants of the quality of learning and success in both face-to-face and virtual learning environments. Both intrinsic or internal and extrinsic or external motivation can drive the students to participate actively in the online learning process. A student's internal motivation largely depends on his/her interest in the topic, self-requirement, self-determination, self-regulation, and autonomy of learning. The external motivation of students is determined by the teacher's behaviour, teaching-learning strategies, and students' engagement [48]. Being a medical teacher, teachers have to consider all these factors and plan the online teaching accordingly. Generate a sense of belongingness among the students and, instead of making a passive learner, involve them in the learning process. Remember and call students by their names, ask provocative questions, encourage them to speak, and appreciate when they attempt to respond. Another effective way to motivate students is to give feedback to the students after each Class's end. Teachers' constructive feedback helps students identify their strengths and weaknesses and motivate them to engage in learning [49]. The teacher should also emphasize peer feedback in the Class because it stimulates active learning [50].

### 4.10. Select appropriate assessment mode

Over the decades, there has been a growing interest in using digital technologies to support learning and assessment [51]. However, research is lacking about the effectiveness of online assessment. The emergence of COVID-19 has further increased significant opportunities to develop new assessment forms [1]. There is a need to develop online assessment tools in medical education that are capable of assessing the cognitive (knowledge), psychomotor (skills), and affective (behaviour/attitude) domains of medical students. Medical educators should carefully consider the assessment tools, ensuring that they align with the immediate learning outcomes. Some of the basic online modes of assessments are knowledge-based assessment (e.g., Multiple choice questions, extended matching questions), performance-based assessment (e.g., Using virtual OSCE station), and practice-based assessment (e.g., Use of logbook and portfolios and attitude based assessment (teachers' observation in the discussion or presentations, peer-assessment) [52].

Kühbeck et al. conducted a study with a cohort of undergraduate medical students at the Technical University of Munich, where students were given access to an online assessment consisting of 440 MCQs after a four-week teaching Pharmacology. They found that online assessments improved the self-perceived pharmacology competence of medical students. Another study conducted on medical students in India suggests the effectiveness of online MCQs assessment. Students responded that online assessment was easy to operate, reducing malpractices, and noticed that they were exposed to innovative methods of learning, and they received immediate results. Gaytan and McEwen conducted a study on effective online instructional and assessment strategies perceived by faculty and students. They found a variety of effective assessment techniques, as perceived by faculty and students, including weekly assignments with immediate feedback, self-assessments, projects, portfolios, peer evaluation, and quizzes [18],[53]. To evaluate the learning outcomes in anatomy, various assessment modes, including essay-type questions, practical examination, multiple-choice questions examination (MCQE), and spatial MCQE, have been helpful.

Kearns (2012) examined 24 online courses and identified assessment methods that include: written assignments (research papers, case studies, and short essays); online discussion (asynchronous discussion activity performed on a discussion board or blog; fieldwork (students write a report after collecting field data; quizzes and exams (Multiple choice questions, short answer questions; and presentations (students' online presentation) [54]. These modes of assessments are relevant to medical and health professionals if these are correctly used. The open-book examination could be a more compelling online assessment method that allows educators to pose questions that require critical thinking and higher-order cognitive skills [55]. Timely feedback is also a critical component of an online assessment. Students should know their progress through the online assessment, and they should be guided where they need improvement.

Despite the variety of assessment tools available, multiple-choice questions play an integral part in assessing students' learning performance face-to-face and in the online mode of examination.

## 5. Conclusions

The COVID-19 crisis has created an opportunity to think about new ways of teaching and learning in medical education. Even after the pandemic, online education will be a significant part of health care delivery. Medical educators must learn from the experience and cultivate competency with technology for future learning needs. Schools should strengthen technical infrastructure, promote continuous faculty development programs, and support indigent students to access digital technology.

The importance of online education in assessing the learning outcomes through various modes is essential for successful academic progress both to teachers and to the students. It will be worthy to study if the anticipated conclusions are achieved after introducing these varying modes and methods to course delivery. The corroboration of benefits, practicality, drawbacks and modifiable drivers of various methods and modes of online education is entailing. If found to be beneficial to the teachers, students and the institution, these methods can be integrated in courses on a long-term basis following the cessation of the pandemic and not just to be limited during the present active COVID-19 period.
